# Views of Implementers and Nonimplementers of Internet-Administered Cognitive Behavioral Therapy for Depression and Anxiety: Survey of Primary Care Decision Makers in Sweden

**DOI:** 10.2196/18033

**Published:** 2020-08-12

**Authors:** Anders Brantnell, Joanne Woodford, Enrico Baraldi, Theo van Achterberg, Louise von Essen

**Affiliations:** 1 Clinical Psychology in Healthcare Department of Women's and Children's Health Uppsala University Uppsala Sweden; 2 Division of Industrial Engineering and Management Department of Civil and Industrial Engineering Uppsala University Uppsala Sweden; 3 KU Leuven Department of Public Health and Primary Care Academic Centre for Nursing and Midwifery Leuven Belgium

**Keywords:** mental health, internet-administered CBT, self-management, implementation, barriers and facilitators, decision-making, eHealth, primary care

## Abstract

**Background:**

Internet-administered cognitive behavioral therapy (ICBT) has been demonstrated to be an effective intervention for adults with depression and/or anxiety and is recommended in national guidelines for provision within Swedish primary care. However, the number and type of organizations that have implemented ICBT within primary care in Sweden is currently unclear. Further, there is a lack of knowledge concerning barriers and facilitators to ICBT implementation.

**Objective:**

The two primary objectives were to identify and describe primary care organizations providing ICBT in Sweden and compare decision makers’ (ie, directors of primary care organizations) views on barriers and facilitators to implementation of ICBT among ICBT implementers (ie, organizations that offered ICBT) and nonimplementers (ie, organizations that did not offer ICBT).

**Methods:**

An online survey based on a checklist for identifying barriers and facilitators to implementation was developed and made accessible to decision makers from all primary care organizations in Sweden. The survey consisted of background questions (eg, provision of ICBT and number of persons working with ICBT) and barriers and facilitators relating to the following categories: users, therapists, ICBT programs, organizations, and wider society.

**Results:**

The participation rate was 35.75% (404/1130). The majority (250/404, 61.8%) of participants were health care center directors and had backgrounds in nursing. Altogether, 89.8% (363/404) of the participating organizations provided CBT. A minority (83/404, 20.5%) of organizations offered ICBT. Most professionals delivering ICBT were psychologists (67/83, 80%) and social workers (31/83, 37%). The majority (61/83, 73%) of organizations had 1 to 2 persons delivering ICBT interventions. The number of patients treated with ICBT during the last 12 months was 1 to 10 in 65% (54/83) of the organizations, ranging between 1 and 400 treated patients across the whole sample. There were 9 significant (*P*<.05) differences out of 37 possible between implementers and nonimplementers. For example, more implementers (48/51, 94%) than nonimplementers (107/139, 76.9%) perceived few technical problems (*P*<.001), and more implementers (53/77, 68%) than nonimplementers (103/215, 47.9%) considered that their organization has resources to offer ICBT programs (*P*<.001).

**Conclusions:**

Despite research demonstrating the effectiveness of ICBT for depression and anxiety and national guidelines recommending its use, ICBT is implemented in few primary care organizations in Sweden. Several interesting differences between implementers and nonimplementers were identified, which may help inform interventions focusing on facilitating the implementation of ICBT.

## Introduction

### Background

According to the World Health Organization, over 300 million people suffer from depression worldwide [1]. Further, over 260 million people suffer from anxiety disorders (eg, generalized anxiety disorder, panic disorder, and social anxiety disorder) [1]. Both depression and anxiety result in individual suffering and are associated with increased costs for both individuals and wider society [2-5]. One evidence-based intervention for depression and anxiety disorders (hereafter anxiety) is cognitive behavioral therapy (CBT), traditionally delivered face to face by a therapist [6-9]. However, despite the evidence base for CBT, a mental health treatment gap remains globally [10,11]. Some reasons for this treatment gap concern the fact that therapy is resource-intensive (eg, needing facilities to meet patients, requiring patients to travel to clinics, and needing trained, competent, and expensive therapists) [12]. One potential solution to overcome this treatment gap is the provision of psychological interventions via information technology and new media, referred to as e-mental health [13,14]. Internet-administered CBT (ICBT) [15-18] is a form of e-mental health that has been demonstrated to be as effective as face-to-face CBT [19] and may represent a cost-effective solution, which may increase the availability of evidence-based psychological interventions [20,21].

Sweden was among the first countries to conduct research on the efficacy of ICBT [16,22-25], leading to the introduction of national guidelines regarding the treatment of depression and anxiety [26]. These national guidelines are directed to decision makers at the level of primary care and offer health care providers with evidence-based intervention recommendations, including the provision of CBT and ICBT [26]. Specifically, national guidelines recommend ICBT to be offered to adults with mild to moderate symptoms of depression and anxiety at the primary care level [26]. The guidelines were recently revised to state health care providers should be able to choose the mode of CBT delivery by themselves, with recommended modes of delivery being individual, group, or internet-administered [27].

However, guideline introduction and uptake is difficult, and guidelines are not implemented into routine care automatically [28-32]. While barriers and facilitators to implementation in health care more generally have been identified and related to patients, informal caregivers, practitioners, health care organizations, and wider society [33], few studies have focused on barriers and facilitators to ICBT implementation more specifically [34-36]. Indeed, studies on ICBT implementation into clinical practice focus mostly on effectiveness and clinical feasibility [20,37-41]. Despite the current literature on ICBT implementation being in its infancy, recent studies have begun to identify barriers and facilitators. For example, evidence supporting ICBT programs is reported to be a key facilitator to implementation [34,35], whereas lack of resources [34] and lack of integration with the mental health care system [36] are reported to be important barriers to ICBT implementation. Implementation of e-mental health has received some interest, and studies have identified barriers such as lack of therapists’ knowledge on e-mental health [42] and lack of evidence-based programs [43]. Further, a recent systematic review of barriers and facilitators to implementation included 47 studies and identified factors such as patient and professional acceptance of e-mental health and fit with existing technologies [44]. In addition, while implementation of e-health interventions more generally is well covered in existing research [45-51], there have been recent calls to place more focus on implementation of e-health [52] to enable health care organizations to benefit from the promises of e-health solutions [53].

Studies examining the implementation of ICBT in clinical practice generally focus on the perspectives of patients and practitioners [20,37,39,41]. More recent studies have also included health care managers [34,36]. Although these three groups are important for successful implementation, these studies do not consider the formal decision to implement, which is usually made by primary care and specialized care directors. Consequently, research considering important stakeholders (ie, decision makers directly affecting the implementation of ICBT and their corresponding health care organizations) is lacking. More precisely, at present there is a lack of knowledge about the opinions of key decision makers regarding barriers and facilitators to implementation and which organizations have implemented ICBT in Sweden. Further, while some research has focused on decision makers operating at systems and national level (ie, policy makers and academic researchers) [43], there is a lack of research on decision makers closer to the implementation context and the health care setting such as primary care organization directors.

### Aims and Objectives

This study has two main objectives: (1) identify and describe the primary care organizations providing ICBT in Sweden and (2) compare decision makers’ (ie, directors of primary care organizations) views on barriers and facilitators for the implementation of ICBT in organizations that have implemented ICBT (ie, are offering ICBT [implementers]) and have not implemented ICBT (ie, are not offering ICBT [nonimplementers]). In implementation research, it is common to distinguish between diffusion (ie, passive spread of innovations), dissemination (ie, active efforts to convince an organization to adopt and innovation), implementation (ie, active efforts to offer an innovation and integrate it within the organization), and sustainability (ie, making an innovation part of routine care) [54]. The Swedish guidelines recommending ICBT are relatively new and thus it is unlikely ICBT would be part of routine care in organizations; the assumption is that those who offer ICBT are in the process of integrating ICBT. To this end, the term implementation is used to describe organizations that offer ICBT.

## Methods

### Study Design

An online self-report survey was conducted between February and May 2016 with decision makers in primary care organizations in Sweden. The Checklist for Reporting Results of Internet e-Surveys [55] was followed.

### Setting

Sweden has 290 municipalities situated across 21 administrative units called regions, which are responsible for health care provision. Each region has several primary care organizations forming the basis of the Swedish health care system. According to the Swedish law on health care (Chapter 2, 6§), primary care is part of the open care system and should provide basic medical treatment, ongoing care, preventive measures, and rehabilitation in cases that do not require medical and technical resources or other specialized competence accessible at hospitals [56]. Primary care is thus the first point of care and from here patients can be referred to specialized care. A typical primary care organization employs medical doctors, nurses, physiotherapists, and psychologists and can thus treat many patients. The number of health care professionals employed at primary care organizations is related to the number of listed patients, which ranges from approximately 3000 to 30,000 per organization. Consequently, some primary care organizations have relatively few listed patients whereas others have very many.

Both private and public primary care organizations receive funding from regions and operate under the same conditions in terms of personnel competence, financial resources, opening hours, patient access, and adherence to national guidelines regarding care provision. For primary care organizations willing to implement ICBT, there are three main options available: (1) buying a license from a company for proprietary ICBT programs and delivering the support by themselves, (2) hiring a company to deliver ICBT including support, or (3) connecting to the Platform for Support and Care. The Platform for Support and Care is owned and run by a company (Inera) owned by the Swedish Association of Local Authorities and Regions (SALAR). The Platform for Support and Care offers ICBT programs developed by companies and/or research groups who in turn receive financial compensation when their programs are used. All programs delivered via the Platform for Support and Care undergo a careful procedure to ensure effectiveness and safety. When connecting to this platform, regions can hire companies to support ICBT or use their own therapists to support ICBT. Connecting to the Platform for Support and Care and gaining access to ICBT programs results in financial costs for the regions, such as costs for connecting to the platform and purchasing treatment programs either with or without therapist support. As such, access to ICBT programs and therapist support may differ depending on the financial resources of the health care organization. Further, access to ICBT comes at a financial cost, often per treatment contact with a therapist. For instance, in Stockholm each contact costs around €10 (US $11.22) per session.

### Recruitment and Study Procedures

Decision makers were heads of Swedish primary care organizations identified via SALAR, which supports development and provision of health care, and the Inspectorate for Treatment and Care, responsible for monitoring Swedish health care. We compiled a list of 1156 primary care organizations and mailing addresses and sent invitations to the decision makers via regular mail (see Multimedia Appendix 1 for an English translation of the invitation). Invitations were sent to the organizations because we did not have a complete list of decision makers’ email addresses. Invitations included a link to the online survey administered using the web-based tool, SurveyMonkey, full study information, and participation number and password allowing potential participants to reach the survey. Informed consent was provided online via SurveyMonkey, after which potential participants could access the survey. No incentives were offered for survey completion.

To maximize response rates, reminders were used [57]. Decision makers not completing the survey within two weeks received up to two telephone reminders and an email (except those whose email addresses were not available). The reminder email consisted of study information, the online link to the survey, participation number and password, and a reminder that the survey should be answered within two weeks. The reminder email was also accompanied by the letter previously sent through regular mail. After two weeks, nonrespondent decision makers were reminded once more following the same procedure.

To avoid duplicate responses from decision makers, the SurveyMonkey function allowing only one answer per computer was selected. Respondents who did not complete the survey at one occasion were able to return to the survey at a later time. The survey could be submitted even if all items were not answered. Approval was granted by the ethical review board that reviews applications from Uppsala University, Sweden (application number 2015/461).

### Measures

**Figure 1:**
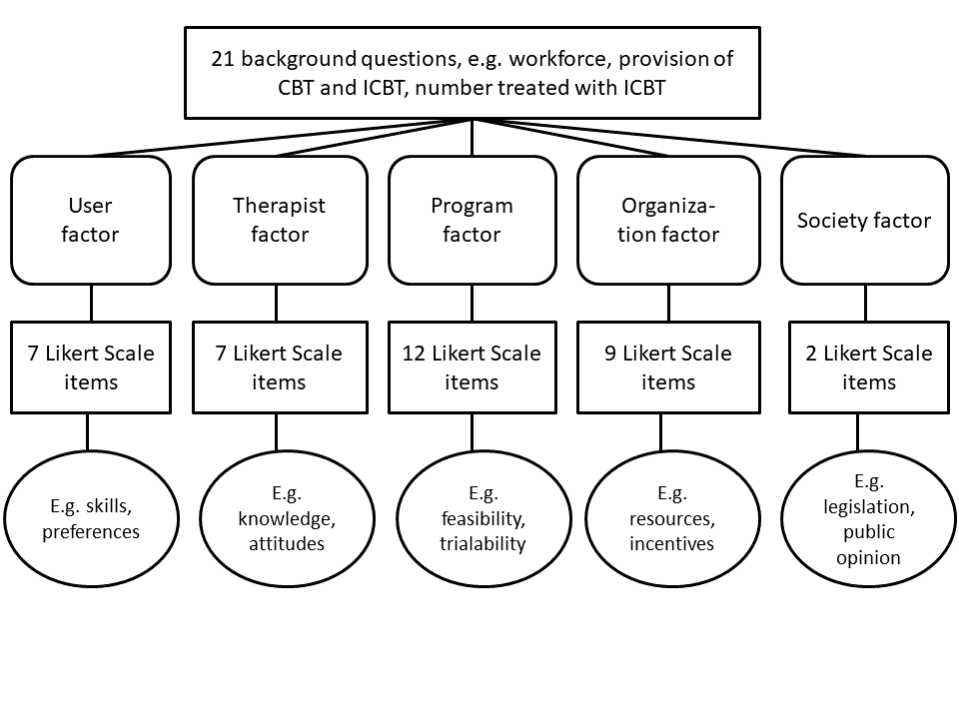
Survey structure.

The study team developed an online survey consisting of 21 background questions and 37 items about 5 factors: (1) user, (2) therapist, (3) program, (4) organization, and (5) society, with a 7-point Likert scale (1 = strongly disagree, 7 = strongly agree) and options for “do not know” and “do not wish to answer.” The survey also included two open-ended questions about barriers and facilitators to implementation and space for additional comments. This qualitative data will be reported elsewhere. The survey was informed by the checklist of barriers and facilitators for improvement of health care practice by Flottorp et al [33] and modified based on knowledge of barriers and facilitators specific to ICBT implementation originating from studies on effectiveness and clinical feasibility available when the study was designed [20,21,38,58-60]. The survey was piloted by a panel of 8 persons with knowledge of ICBT who were not part of the study population, including former primary care directors and persons responsible for information technology and health care in certain Swedish regions. The SALAR database was used to recruit the panel. Panel members were asked to indicate whether the questions were easy to understand and to provide suggestions for improvements. Overall, panel members were positive toward survey content but had some suggestions for improvements (see Multimedia Appendix 2 for a detailed description of the development and a summary of the changes). Figure 1 displays the final overall survey structure, and Multimedia Appendix 3 provides an English translation of the survey.

### Statistical Analysis

All calculations were performed in R version 3.5.2 (R Foundation for Statistical Computing).

#### Responders, Survey Reminders, and Reasons for Not Answering

Descriptive statistics were used to describe numbers and proportions concerning survey completeness, reminders, and reasons for not answering.

#### Characteristics of Study Sample and Organizational Characteristics

Study sample and organizational characteristics are described using descriptive statistics.

#### Representativeness of the Study Sample

As noncoverage (ie, the survey fails to include some parts of the population [61]) is common in survey research, the coverage of the obtained sample was examined. Data from three known background variables for the entire population were used (ie, organizational form, city size, and health care region) and described through descriptive statistics. A chi-square test for equality of proportions was used to examine the representativeness of the sample in terms of the entire population.

#### Barriers and Facilitators to Implementation of Internet-Administered Cognitive Behavioral Therapy

The potential difference regarding the number of agree answers between implementers and nonimplementers was examined using the chi-square test for equality of proportions. The chi-square tests included both the number of nonagree (Likert scale options 1, 2, 3, and 4) and agree (Likert scale options 5, 6, and 7) answers. It was assumed that if a decision maker answered that the organization did not offer CBT (Q6), they did not offer ICBT (Q12), and the respondent was therefore transferred directly from Q6 to Q22. To this end, nonimplementation was imputed for Q12 for the decision makers who answered that they did not offer CBT (Q6).

## Results

### Responders

A total of 1156 survey invitations were sent, of which 97.75% (1130/1156) were shown to be eligible. Noneligible answers that were excluded were duplicate answers (n=13), bankruptcy/closed down (n=10), and not a primary care organization (n=3). A total of 426 decision makers answered at least the first survey question, providing an indication of interest to participate rate of 37.69% (426/1130). Participation [43] was defined as answering questions 1 to 22 (ie, background questions). The participation rate was 35.75% (404/1130). Completeness [55] was defined as completion of the last item (Q58). The completeness rate was 31.94% (361/1130).

### Reminders

A total of 5.4% (22/404) of participants (based on participation rate) responded without a reminder, 47.5% (192/404) responded after being reminded via phone and email, 46.5% (188/404) responded after being reminded via email, and 0.4% (2/404) could not be reminded.

### Reasons for Not Responding

Of the decision makers who did not answer the survey, 17.6% (128/726) provided reasons for not responding. The most frequent reason was no time (n=67), followed by not relevant for us (n=17) and decision maker employment ending (n=14).

### Characteristics of the Study Sample and Organizational Characteristics

The majority (250/404, 61.8%) of decision makers were health care center directors with a background in nursing. A total of 89.8% (363/404) of organizations provided CBT, and 30.1% (122/404) had tried ICBT. Only 20.5% (83/404) reported that they currently offered ICBT and were accordingly categorized as ICBT implementers. Among the professionals delivering ICBT, 80% (67/83) were psychologists (including intern psychologists and psychotherapists) and 37% (31/83) were social workers. The majority (61/83, 73%) of organizations had 1 to 2 persons delivering ICBT. ICBT was offered to 1 to 10 patients during the last 12 months in 65% (54/83) of the organizations. Only 2% (2/83) of organizations had offered ICBT to over 100 persons during the last 12 months. Access to ICBT seemed to depend upon referral by a CBT therapist (36/83, 43%) or general practitioner (25/83, 30%) within the organization (for details on all collected characteristics see Multimedia Appendix 4).

### Representativeness of the Sample

To evaluate the coverage of the sample, data on three known demographic variables for the entire population of primary care organizations in Sweden were used: organizational type (public/private), localization in cities of different sizes (city size), and health care regions. Table 1 displays the distribution of these variables in the study sample and entire population. Data indicates the study sample includes different organizational forms, city sizes, and health care regions. There was a significant difference between distribution of respondents and nonrespondent in terms organizational form (*P*<.001) and health care region (*P*<.001). There was no significant difference between distribution of respondents and nonrespondents regarding city size (*P*=.24). Consequently, the sample was representative in terms of city size but not concerning organizational form and health care region.

**Table 1:** Distribution of respondents in study sample and population.

Demographic		Sample (n=404), n (%)	Population, n/N (%)
**Organizational form**			
	Private	131 (32.4)	131/465 (28.1)
	Public	273 (67.5)	273/665 (41.0)
**City size**			
	Small (<30,000)	148 (36.6)	148/382 (38.7)
	Medium (30,000-100,000)	148 (36.6)	148/418 (35.4)
	Large (>100,000)	108 (26.7)	108/330 (32.7)
**Health care region^a^**			
	North	61 (15.0)	61/123 (49.5)
	Uppsala-Örebro	85 (21.0)	85/231 (36.7)
	Stockholm	64 (15.8)	64/208 (30.7)
	West-East	57 (14.1)	57/130 (43.8)
	South	68 (16.8)	68/222 (30.6)
	West	69 (17.0)	69/216 (31.9)

^a^In Sweden, 21 regions are organized into 6 health care regions that facilitate cooperation and strategic work between the 21 regions.

### Barriers and Facilitators to Implementation of Internet-Administered Cognitive Behavioral Therapy

#### Likert Scale Options and Item Nonresponse

Tables 2 to 6 display the numbers and percentages of decision makers, both implementers and nonimplementers, who agreed (Likert scale options 5, 6, and 7) with items Q22 through Q58. The survey options “do not know” and “do not wish to answer” are treated as item nonresponse and also shown in Tables 2 to 6 [61]. Item nonresponse varied from 22% to 66%.

**Table 2:** Implementers and nonimplementers agreeing to user-related items.

Variable	Item agreement and nonresponse			
	No ICBT^a^ agree n/sample size (%)	ICBT agree n/sample size (%)	*P* value	Item nonresponse^b^ (n=403)
Q22. Adults with depression and/or anxiety have the computer skills needed to use ICBT programs.	177/236 (75.0)	42/62 (67.7)	.32	105 (26.0)
Q23. Adults with depression and/or anxiety are capable of working on their own with ICBT programs.	151/217 (69.5)	39/59 (66.1)	.72	127 (31.5)
Q24. Adults with depression and/or anxiety have interest in ICBT programs.	71/156 (45.5)	29/60 (48.3)	.86	188 (46.6)
Q25. Adherence increases when treatment is delivered online to adults with depression and/or anxiety.	46/113 (40.7)	16/36 (44.4)	.84	254 (63.0)
Q26. The barrier to seek help for adults with depression and/or anxiety is decreased when care is online.	123/183 (67.2)	19/37 (51.3)	.10	183 (45.4)
Q27. Adults with depression and/or anxiety prefer to give confidential information to a computer.	51/144 (35.4)	7/35 (2.0)	.12	224 (55.5)
Q28. Adults with depression and/or anxiety in rural areas can be reached with ICBT programs.	208/243 (85.5)	53/64 (82.8)	.72	96 (23.8)

^a^ICBT: internet-administered cognitive behavioral therapy.

^b^Item nonresponse (do not know, do not wish to answer) of the entire sample (n=403).

**Table 3:** Implementers and nonimplementers agreeing to therapist-related items.

Variable	Item agreement and nonresponse			
	No ICBT^a^ agree n/sample size (%)	ICBT agree n/sample size (%)	*P* value	Item nonresponse^b^ (n=396)
Q29. Therapists treating adults with depression and/or anxiety are positive toward the ICBT programs.	108/166 (65.0)	57/71 (80.2)	.03	159 (40.1)
Q30. Therapists treating adults with depression and/or anxiety have knowledge of the ICBT programs.	125/192 (65.1)	68/76 (89.4)	.001	128 (32.3)
Q31. Therapists treating adults with depression and/or anxiety only need a little training in ICBT programs.	94/140 (67.1)	31/52 (59.6)	.42	204 (51.5)
Q32. Therapists treating adults with depression and/or anxiety have the computer skills needed for ICBT.	195/227 (85.9)	66/72 (91.6)	.28	97 (24.4)
Q33. Therapists treating adults with depression and/or anxiety have confidence in the guidelines recommending ICBT programs.	103/148 (69.5)	55/62 (88.7)	.001	186 (46.9)
Q34. Therapists treating adults with depression and/or anxiety can motivate patients to participate.	159/199 (79.8)	54/66 (81.8)	.87	131 (33.0)
Q35. More therapists support the introduction of ICBT programs than oppose it.	89/119 (74.7)	40/49 (81.6)	.45	228 (57.5)

^a^ICBT: internet-administered cognitive behavioral therapy.

^b^Item nonresponse (do not know, do not wish to answer) of the entire sample (n=396).

**Table 4:** Implementers and nonimplementers agreeing to program-related items.

Variable	Item agreement and nonresponse			
	No ICBT^a^ agree n/sample size (%)	ICBT agree n/sample size (%)	*P* value	Item nonresponse^b^ (n=389)
Q36. ICBT programs for adults with depression and/or anxiety should come with a help desk for therapists.	164/198 (82.8)	41/45 (91.1)	.25	146 (37.5)
Q37. ICBT programs are well suited for adults with depression and/or anxiety.	142/184 (77.1)	52/62 (83.8)	.37	143 (36.7)
Q38. ICBT programs for adults with depression and/or anxiety offer alternative learning formats.	77/101 (76.2)	15/32 (46.8)	.001	256 (65.8)
Q39. ICBT programs for adults with depression and/or anxiety are not plagued with big technical problems.	107/139 (76.9)	48/51 (94.1)	.001	199 (51.1)
Q40. It should be possible to trial the ICBT programs.	222/247 (89.8)	45/55 (81.8)	.15	87 (22.3)
Q41. It is possible to measure the effect on depression and/or anxiety when providing ICBT programs.	122/137 (89.0)	36/42 (85.7)	.75	210 (53.9)
Q42. ICBT programs for adults with depression and/or anxiety are easy to use.	78/95 (82.1)	46/52 (88.4)	.44	242 (62.2)
Q43. ICBT programs for adults with depression and/or anxiety can be integrated with the care structure.	161/208 (77.4)	63/73 (86.3)	.15	108 (27.7)
Q44. ICBT programs for adults with depression and/or anxiety can replace face-to-face CBT.	58/202 (28.7)	18/62 (29.0)	.99	125 (32.1)
Q45. It is easy to get access to ICBT programs for adults with depression and/or anxiety.	59/111 (53.1)	39/57 (68.4)	.08	221 (56.8)
Q46. ICBT programs for adults with depression and/or anxiety are well grounded on research evidence.	95/117 (81.1)	39/49 (79.5)	.98	223 (57.3)
Q47. GPs^c^ referring adults with depression and/or anxiety to ICBT are positive toward ICBT programs.	88/130 (67.6)	30/46 (65.2)	.90	213 (54.7)

^a^ICBT: internet-administered cognitive behavioral therapy.

^b^Item nonresponse (do not know, do not wish to answer) of the entire sample (n=389).

^c^GPs: general practitioners.

**Table 5:** Implementers and nonimplementers agreeing to organization-related items.

Variable	Item agreement and nonresponse			
	No ICBT^a^ agree n/sample size (%)	ICBT agree n/sample size (%)	*P* value	Item nonresponse^b^ (n=381)
Q48. Our organization has resources to offer ICBT programs to adults with depression and/or anxiety.	103/215 (47.9)	53/77 (68.8)	.001	89 (23.3)
Q49. ICBT programs can decrease care costs in treatment of adults with depression and/or anxiety.	157/196 (80.1)	42/57 (73.6)	.39	128 (33.5)
Q50. Existing information management systems allows administration of patients enrolled in ICBT.	57/105 (54.2)	34/54 (62.9)	.38	222 (58.2)
Q51. Existing quality assurance and patient safety systems are compatible with the requirements to offer ICBT to adults with depression and/or anxiety.	56/85 (65.8)	35/44 (79.5)	.16	252 (66.1)
Q52. Existing continuing education systems of therapists are compatible with the training to introduce ICBT to adults with depression and/or anxiety.	58/91 (63.7)	31/44 (70.4)	.56	246 (64.5)
Q53. Internal regulations allow introduction of ICBT for adults with depression and/or anxiety.	123/152 (80.9)	64/71 (90.1)	.12	158 (41.4)
Q54. Contracts with service providers allow introduction of ICBT for adults with depression and/or anxiety.	59/89 (66.2)	41/47 (87.2)	.02	245 (64.3)
Q55. The concept of online treatment to adults with depression and/or anxiety is well established at our organization.	35/226 (15.4)	36/72 (50.0)	.001	83 (21.7)
Q56. The patient referral process allows introduction of ICBT for adults with depression and/or anxiety.	87/164 (53.0)	58/66 (87.8)	.001	151 (39.6)

^a^ICBT: internet-administered cognitive behavioral therapy.

^b^Item nonresponse (do not know, do not wish to answer) of the entire sample (n=381).

**Table 6:** Implementers and nonimplementers agreeing to society-related items.

Variable	Item agreement and nonresponse			
	No ICBT^a^ agree n/sample size (%)	ICBT agree n/sample size (%)	*P* value	Item nonresponse^b^ (n=361)
Q57. Legislation does not hinder the introduction of ICBT for adults with depression and/or anxiety.	128/143 (89.5)	48/53 (90.5)	.99	165 (45.7)
Q58. Public opinion supports the introduction of online treatments for adults with depression and/or anxiety.	91/123 (73.9)	26/37 (70.2)	.81	201 (55.6)

^a^ICBT: internet-administered cognitive behavioral therapy.

^b^Item nonresponse (do not know, do not wish to answer) of the entire sample (n=361).

#### Users

There were no significant differences (*P*<.05) between implementers and nonimplementers in terms of agreeing to user-related items (Table 2).

#### Therapists

Three therapist-related items were significant (*P*<.05; Table 3). More implementers (57/71, 80%) than nonimplementers (108/166, 65.0%) believed that therapists treating adults with depression and/or anxiety are positive toward the ICBT programs (*P*=.03; Q29). More implementers (68/76, 89%) than nonimplementers (125/192, 65.1%) believed therapists treating adults with depression and/or anxiety have knowledge of the ICBT programs (*P*<.001; Q30). More implementers (55/62, 88%) than nonimplementers (103/148, 69.5%) also believed therapists treating adults with depression and/or anxiety have confidence in the guidelines recommending ICBT programs (*P*<.001; Q33).

#### Program

Two program-related items were significant (*P*<.05; Table 4). More nonimplementers (77/101, 76.2%) than implementers (15/32, 46%) believed that ICBT programs for adults with depression and/or anxiety offer alternative learning formats (*P*<.001; Q38). More implementers (48/51, 94%) than nonimplementers (107/139, 76.9%) in turn believed that ICBT programs for adults with depression and/or anxiety are not plagued with big technical problems (*P*<.001; Q39).

#### Organization

Four organization-related items were significant (*P*<.05; Table 5). More implementers (53/77, 68%) than nonimplementers (103/215, 47.9%) considered that they have resources to offer ICBT programs (*P*<.001; Q48). More implementers (41/47, 87%) than nonimplementers (59/89, 66%) believed that contracts with service providers allow introduction of ICBT (*P*=.02; Q54). More implementers (36/72, 50%) than nonimplementers (35/226, 15.4%) considered that the concept of online treatment is well established at their organization (*P*<.001; Q55). More implementers (58/66, 87%) than nonimplementers (87/164, 53.0%) believed the patient referral process allows for the introduction of ICBT (*P*<.001; Q56).

#### Society

There were no significant differences (*P*<.05) between implementers and nonimplementers in terms of society-related items (Table 6).

## Discussion

### Principal Findings

The participation rate was 35.75% (404/1130). The majority (250/404, 61.8%) of participants were health care center directors with a background in nursing. A total of 89.9% (363/404) of the participating organizations provided CBT. A minority (83/404, 20.5%) provided ICBT and thus were implementers. In general, psychologists (67/83, 80%) and social workers (31/83, 37%) delivered ICBT to patients, and the majority (61/83, 73%) of the organizations had 1 to 2 persons delivering ICBT. There were 9 significant (*P*<.05) differences out of 37 possible between implementers and nonimplementers. For example, more implementers (68/76, 89%) than nonimplementers (125/192, 65.1%) considered that therapists treating adults with depression and/or anxiety have knowledge of the ICBT programs (*P*<.001), and more implementers (58/66, 87%) than nonimplementers (87/164, 53.0%) believed the patient referral process allows the introduction of ICBT (*P*<.001).

Although e-mental health initiatives are encouraged, and the Swedish national guidelines include provision of ICBT in Swedish primary care among their recommendations, few organizations in our sample provided ICBT. Moreover, in a majority of cases only 1 to 10 patients had been treated with ICBT during the last 12 months, and the majority of organizations only had 1 to 2 CBT therapists working with ICBT. The number of ICBT implementers is not as high as one would expect based on the strong recommendations and claimed benefits of ICBT, such as increased patient access and lower costs [21,22]. Previous research indicates the implementation rate of guidelines across various treatment areas has been between 50% and 70% [62-65]. However, implementation research does not provide detailed guidance concerning whether a 20% implementation rate could be considered low or high. Further, the implementation of a complex health care intervention, such as ICBT, is more demanding than implementing clinical guidelines in general. Indeed, our findings are in line with a recent study examining ICBT implementation among psychologists in the Netherlands where the implementation rate was around 16% [66].

One reason for the low implementation rate may pertain to not having a workforce who can support the provision of ICBT. Indeed, the majority of implementing organizations had only 1 to 2 therapists working with ICBT and CBT. Moreover, all organizations providing ICBT also provided CBT, and in cases ICBT and CBT rely on the same workforce, this workforce could be inadequate to support ICBT implementation. Further, CBT therapists may not represent the best solution, given they are a highly trained and expensive workforce, with a demand exceeding supply [67]. A possible solution could be to train mental health workers to support ICBT, as done by the Improving Access to Psychological Therapies program in England, whereby a workforce of psychological well-being practitioners was established to deliver low-intensity CBT [67]. Future research could explore the workforce aspect in Sweden, and current findings could be helpful for policymakers trying to promote the implementation of ICBT.

Our findings show that 94% (48/51) of implementers consider that ICBT is not plagued with technical problems, whereas only 76.9% (107/139) of nonimplementers share this opinion (Q39). Existing research on implementation is clear regarding technical problems, showing innovations low in complexity to be more easily adopted [68-70]. Another interesting finding is that 50% (36/72) of implementers believed that the concept of online treatment regarding depression and/or anxiety is well established at their organization compared with 15.4% (35/226) of nonimplementers who believed this be the case (Q55). The survey did not define the word concept, and thus this could have been interpreted differently by decision makers. However, as relatively many implementers compared with nonimplementers perceived the concept to be well established there seems to be a clear pattern in how this item is perceived by the decision makers. One possible reason for this finding could be that implementation of ICBT may increase understanding of online treatments and thus strengthen the concept.

Another interesting finding is that the majority of implementers (53/77, 68%) consider that they have resources to offer ICBT programs compared with a minority among nonimplementers (103/215, 47.9%; Q48), which indicates lack of resources may be an important barrier to implementation of ICBT in Swedish primary care. This factor is identified as central in existing ICBT implementation research [34] and in research concerning implementation of eHealth [45,71]. Findings also show that 80% (57/71) of implementers consider therapists treating adults with depression and/or anxiety are positive toward the ICBT programs compared with 65.0% (108/166) of nonimplementers (Q29). This finding implies therapist acceptance could be an important facilitator to implementation, in line with existing ICBT implementation research [21,60] and research concerning e-mental health [42,44]. Findings also demonstrate that 87% (41/47) of implementers believe contracts with service providers allow for the introduction of ICBT compared with 66% (59/89) of nonimplementers (Q54). Indeed, contracts with service providers are included in the framework regarding barriers and facilitators to implementation by Flottorp et al [33]. As such, our findings may imply lack of contracts with service providers could be a sizeable barrier to implementation of ICBT in Sweden.

While 28 barriers and facilitators to implementation did not show significant differences between implementers and nonimplementers, they still provide important findings. The majority (219/298, 73.4%) of decision makers considered users to possess the computer skills needed to use ICBT programs. Further, the majority (261/299, 87.2%) considered therapists to have the computer skills required to offer ICBT. Lack of computer skills among patients and therapists was found to be a barrier to implementation in wider research concerning implementation of e-mental health [43]. Further, therapists’ computer skills is reported to be a facilitator to implementation of eHealth [45]. Findings also show that the majority (134/166, 80.7%) of decision makers considered ICBT programs to be evidence-based. This factor is a key facilitator in ICBT implementation [34,35] and an important barrier, if absent, in e-mental health [43] and eHealth implementation [47,48]. Further, the majority (199/253, 78.6%) of decision makers considered that ICBT can decrease costs related to providing care, which is also suggested in existing studies concerning eHealth programs [46,47].

### Limitations

First, this study aimed to cover all primary care organizations in Sweden. However, recruitment of decision makers was difficult and thus the study only includes a limited sample of primary care organizations. However, participation rate was 35.75% (404/1130), which is in line with a recent study examining the implementation of ICBT from therapists’ point of view [66] and the sample was representative of the population on city size. Second, we cannot exclude the possibility that people who were interested in ICBT were more inclined to respond and thus our sample may include a larger proportion of implementers than nonimplementers compared with the entire population. Third, we focused on decision makers in Swedish primary care, who are often not experts in ICBT. However, decision makers decide whether or not to implement and thus their perceptions of barriers and facilitators are key for understanding the chances of successful implementation. Future studies may also include the views of Swedish primary care therapists representing implementing organizations and could compare the views of therapists with those of decision makers. Fourth, this study presents results of a survey focusing on a set of factors and whether respondents agreed on their importance on a quantitative scale but not to what extent each factor represented a barrier or facilitator to the implementation process. Future studies combining both survey and semistructured interview data may provide a more detailed exploration of barriers and facilitators to implementation and aid interpretation of survey findings.

Fifth, the survey did not explore whether implementing organizations used specific implementation strategies associated with improved changes to existing practice [29]—for example, opinion leaders or audit and feedback to facilitate implementation [72]. Nor did the survey explore to what extent decision makers considered ICBT implementation a success (eg, in terms of increased ICBT use, costs, and resources). Future research into barriers and facilitators of ICBT implementation may examine ICBT implementation strategies used and the perceived success of these strategies. Sixth, our results are influenced by multiple testing: with alpha .05 and 37 tests, 2 of the 9 differences are likely to be produced by chance alone. Seventh, nonresponse per item was relatively high (varied between 22% and 66%), which could impact the validity of the results. However, we observe that nonresponse is relatively evenly distributed between items and thus a reasonable assumption is that nonresponse does not impact validity.

Despite the aforementioned limitations, to our knowledge, this is the first study to provide data on the implementation of ICBT in one of the countries that pioneered ICBT research and treatment, elucidating the views of decision makers on the implementation of ICBT in primary care.

### Conclusions

Despite existing scientific evidence supporting implementation of ICBT in primary care and guidelines recommending implementation, most primary care organizations in Sweden still only offer traditional CBT. This study provides an overview of the characteristics of implementers in one of the countries that pioneered research in ICBT, identifying interesting differences in terms of perceived barriers and facilitators between implementers and nonimplementers that may inform future implementation interventions to improve the routine uptake of ICBT in Swedish primary care but also in other countries introducing ICBT.

## Appendix

Multimedia Appendix 1 English translation of invitation letter used to recruit respondents.

Multimedia Appendix 2 Survey development.

Multimedia Appendix 3 English translation of the online questionnaire as it was presented to the respondents.

Multimedia Appendix 4 Characteristics of the study sample and organizational characteristics.

## Abbreviations

CBT: cognitive behavioral therapy
ICBT: internet-administered cognitive behavioral therapy
SALAR: Swedish Association of Local Authorities and Regions
