# Supplementary material for: Views of Implementers and Nonimplementers of Internet-Administered Cognitive Behavioral Therapy for Depression and Anxiety: Survey of Primary Care Decision Makers in Sweden
Source: J Med Internet Res. 2020 Aug 12;22(8):e18033. doi: 10.2196/18033 (PMC7450364; doi:10.2196/18033)
Supplement: Multimedia Appendix 1 [file jmir_v22i8e18033_app1.docx]

## **Appendix 1. Invitation letter**

#### Research study: ”barriers and facilitators for the implementation of internet-based cognitive behavioral therapy for adult patients with mild and moderate depression and/or anxiety”

Would you like to contribute to increasing the knowledge concerning the introduction of internet-based cognitive behavioral therapy?

# What is the study about?

Research has shown that both face-to-face cognitive behavioral therapy (CBT) and internet-based cognitive behavioral therapy (ICBT) can help adult patients with mental health problems such as depression and anxiety. Introduction of new treatments often takes a long time and specifically for the introduction of ICBT there are few studies that have looked into this. Moreover, there is limited knowledge concerning the degree to which adult patients with mild and moderate depression and/or anxiety are offered ICBT.

The current study aims to identify and describe organizations that have or have not introduced ICBT and tries to identify possible explanations for introduction vs. non-introduction through exploring barriers and facilitators for ICBT introduction.

# Who can participate and what is the procedure?

The study targets persons like you who have a leading position in working with CBT or ICBT in your organization. If you are interested in participating, we ask you to fill in an online questionnaire by following this link: (link was provided here). The questionnaire deals with the use of CBT and ICBT in your organization and reasons for using or not using ICBT. We ask you to answer at latest the…(two weeks response time was given).

# What about privacy?

All information you provide by filling out the questionnaire will be analyzed anonymously. In presenting and reporting the results, you and your organization will not be recognizable to anyone at any time.

# Are there any risks?

No obvious risks in participating in the study exist.

# Your participation is voluntary and you can withdraw from the study any time

To participate in the study is voluntary and you can withdraw from the study any time without giving a reason for this. Only those you work with the study know who participate.

# Head of research and responsible researcher

The current study is performed in primary care in Sweden.

XX will serve as head of research and with XX as the responsible researcher.
“Contact information provided”

# How do I get information about the study’s results?

Results will be published in international scientific journals. You as a participant can also receive information about the results by sending an e-mail to the responsible researcher.

# In case of questions

If you have questions regarding the study please do not hesitate to contact one of the researchers in the project (see above).

#### yours sincerely

**XX**
