# Supplementary material for: Views of Implementers and Nonimplementers of Internet-Administered Cognitive Behavioral Therapy for Depression and Anxiety: Survey of Primary Care Decision Makers in Sweden
Source: J Med Internet Res. 2020 Aug 12;22(8):e18033. doi: 10.2196/18033 (PMC7450364; doi:10.2196/18033)
Supplement: Multimedia Appendix 2 [file jmir_v22i8e18033_app2.docx]

## **Appendix 2. Survey development**

### The process

The Flottorp et al. [1] checklist was used to lay a foundation for survey development. The Flottorp et al. [1] checklist builds on a systematic review of barriers and facilitators to implementation comprising 87 papers and reviewing 12 existing models and frameworks on barriers and facilitators [1]. The checklist comprises 57 items and seven factors; (1) guideline, (2) health professional, (3) patient, (4) professional interaction, (5) incentives and resources, (6) capacity for organizational change, and (7) social, political and legal factors [1]. As the checklist tries to combine the evidence and factors from all the existing models [1], we perceived it as the state of the art knowledge regarding the barriers and facilitators at the time when our study was designed. However, for our population adopting the full Flottorp et al. [1] checklist was not optimal. Based on our communication with SALAR, primary care decision makers would not have time to answer such a long questionnaire and furthermore the full Flottorp checklist is not tailored to ICBT implementation. As such, a survey was developed based on the Flottorp checklist and available research concerning barriers and facilitators to ICBT implementation.

The online survey was developed in three stages. First, we summarized all barriers and facilitators raised in ICBT implementation research available at the time for study design [2–7] and compared frequently raised barriers and facilitators with ones in the Flottorp et al. [1]checklist. Based on this we included the frequently mentioned barriers and facilitators in previous ICBT research that were also part of the Flottorp et al. [1] checklist. Second, we searched for barriers and facilitators that were not identified in previous ICBT implementation research but were part of the Flottorp et al. [1] checklist and well known barriers and facilitators in implementation research. Third, we searched for barriers and facilitators that were not included in the Flottorp et al. [1] checklist but were frequently mentioned in previous ICBT implementation literature (Table 1 provides the details on survey development)

| **Table 1. Survey development: barriers and facilitators in Flottorp et al. checklist and ICBT implementation research** | | | | | |
| --- | --- | --- | --- | --- | --- |
| Barriers and facilitators | Barriers and facilitators categorized into five different factors | | | | |
|  | Patient | Therapist | Program | Organization | Society |
| Knowledge | Q22 (C^a^) | Q30 (C, ICBT), Q32 (C) |  |  |  |
| Skills | Q22 (C), Q23 (C) | Q31 (C, ICBT), Q32 (C), 34 (C, ICBT) |  |  |  |
| Attitudes | Q24 (C, ICBT^b^) | Q29 (C, ICBT), Q33 (C, ICBT), Q35 (C, ICBT) |  |  |  |
| Treatment adherence | Q25 (C, ICBT) |  |  |  |  |
| Motivation | Q26 (C, ICBT) |  |  |  |  |
| Preferences | Q27 (C, ICBT) |  |  |  |  |
| Accessibility | Q28 (ICBT) |  | Q45 (C) |  |  |
| Program support |  |  | Q36 (C) |  |  |
| Feasibility |  |  | Q37 (C, ICBT), Q38 (ICBT) |  |  |
| Technical problems |  |  | Q39 (ICBT) |  |  |
| Trialability |  |  | Q40 (C) |  |  |
| Visibility |  |  | Q41 (C, ICBT) |  |  |
| Usability |  |  | Q42 (C, ICBT) |  |  |
| Compatibility |  |  | Q43 (C, ICBT) |  |  |
| Replicability |  |  | Q44 (ICBT) |  |  |
| Grounded on research |  |  | Q46 (C, ICBT) |  |  |
| General practitioners’ view |  |  | Q47 (C, ICBT) |  |  |
| Resources |  |  |  | Q48 (C, ICBT) |  |
| Financial incentives |  |  |  | Q49 (C, ICBT) |  |
| Information system |  |  |  | Q50 (C) |  |
| Quality assurance and patient safety |  |  |  | Q51 (C) |  |
| Continuing education system |  |  |  | Q52 (C) |  |
| Internal regulations |  |  |  | Q53 (C) |  |
| Existing contracts |  |  |  | Q54 (C) |  |
| Organizational culture |  |  |  | Q55 (C) |  |
| Referral process |  |  |  | Q56 (C) |  |
| Legislation |  |  |  |  | Q57 (C) |
| Public opinion |  |  |  |  | Q58 (C) |
| ^a^ C refers to Flottorp et al. checklist  ^b^ ICBT refers to ICBT implementation research | | | | | |

### Piloting

The online survey was trialed with a pilot panel consisting of eight respondents. We asked respondents to indicate whether or not the questions were easy to understand and to provide detailed feedback if questions were not easy to understand. Overall respondents were positive towards the survey and its content but they had some suggestions for improvements, which are described below.

**(1) One background question was added concerning the organizational position of the respondent: “What is your position in the organization?”**

**(2) The two questions below (a and b) were combined into one question (c):**

(a) Has your organization bought the ICBT programs that you offer to adults with depression and/or anxiety? (Yes/No)

(b) Has your organization developed the ICBT programs that you offer to adults with depression and/or anxiety? (Yes/No)

*(c) How has your organization received access to the ICBT programs? (We have bought the programs/We have developed the programs/Some other way, how?)*

**(3) The word “program” was added after the word ICBT throughout the survey.**

**(4) The introduction texts to each item questions were adjusted to ensure it was clear that we were asking for the respondents opinions. We also added one additional reply alternative “Do not wish to answer,” throughout the item questions. The texts below illustrate the changes where text “a” is the old text and “b” is the adjusted text:**

(a) Below you will find a set of statements. Please indicate the extent to which you agree/disagree with each statement. 1 = I strongly disagree and 7 = I strongly agree. In case needed you can also choose “Do not know.”

(b) Below you will find a set of statements. Please indicate, based on your judgement, the extent to which you agree/disagree with each statement. 1 = I strongly disagree and 7 = I strongly agree. In case needed you can even choose one of the options; “Do not know” or “Do not wish to answer.” Please note that there are no right or wrong answers.

**(5) The wording “patients” was replaced with “Adults with depression and/or anxiety” throughout the item questions.**

**(6) The wording “psychological problems” was replaced with adults with depression and/or anxiety in one question. The texts below illustrate the changes where text “a” is the old text and “b” is the adjusted text:**

(a) The barrier to seek help for psychological problems is decreased when care is provided through internet.

(b) The barrier to seek help, for adults with depression and/or anxiety, is decreased when care is provided through internet.

**(7) The following wording was added to the questions concerning CBT therapists after the word CBT therapist: “who treat adults with depression and/or anxiety.” The texts below provide an example of the changes where text “a” is the old text and “b” is the adjusted text:**

(a) CBT-therapist has knowledge of ICBT.

(b) CBT therapists’ who treat adults with depression and/or anxiety have knowledge of the ICBT programs.

**(8) The wording ”for adults with depression and/or anxiety” was added in one question. The texts below illustrate the changes where text “a” is the old text and “b” is the adjusted text:**

(a) It is possible to measure the effect on depression and/or anxiety when providing treatment through the ICBT programs.

(b) It is possible to measure the effect on depression and/or anxiety when providing treatment through the ICBT programs for adults with depression and/or anxiety.

**(9) The wording ”targeting adults with depression and/or anxiety” was added in one question.** **The texts below illustrate the changes where text “a” is the old text and “b” is the adjusted text:**

(a) Our organization’s existing patient referral process allows the introduction of the ICBT programs.

(b) Our organization’s existing patient referral process allows the introduction of the ICBT programs targeting adults with depression and/or anxiety.

**(10) Two of the questions concerning “society” were removed altogether as they were considered to be too complicated for health center directors to answer:**

“I consider that country specific ethical regulations concerning internet-based ICBT concerning therapist patient interaction enables introduction of ICBT for adults with depression and/or anxiety”

”According to my understanding political stability enables the introduction of internet-based ICBT for adults with depression and/or anxiety.”

**(11) The wording ”existing laws enable” was replaced by the ”Swedish legislation does not hinder” in one question.** **The texts below illustrate the changes where text “a” is the old text and “b” is the adjusted text:**

(a) Existing laws enable introduction of ICBT to adults with depression and/or anxiety.

(b) The Swedish legislation does not hinder the introduction of ICBT programs to adults with depression and/or anxiety.

**(12) The wording ”in general” was removed and ”targeting adults with depression and/or anxiety” was added in one question.** **The texts below illustrate the changes where text “a” is the old text and “b” is the adjusted text:**

(a) The public opinion in general supports the introduction of internet-based treatments.

(b) The public opinion supports the introduction of internet-based treatments targeting adults with depression and/or anxiety.

## References

1. Flottorp SA, Oxman AD, Krause J, Musila NR, Wensing M, Godycki-Cwirko M, et al. A checklist for identifying determinants of practice: a systematic review and synthesis of frameworks and taxonomies of factors that prevent or enable improvements in healthcare professional practice. Implement Sci. 2013;8(35). doi:10.1186/1748-5908-8-35. PMID:23522377

2. Andersson G, Titov N. Advantages and limitations of Internet-based interventions for common mental disorders. World Psychiatry. 2014;13(1):4–11. doi: [10.1002/wps.20083](https://doi.org/10.1002/wps.20083). PMID:24497236

3. Andersson G. The promise and pitfalls of the internet for cognitive behavioral therapy. BMC Med. 2010 Jan;8(82). doi:10.1186/1741-7015-8-82. PMID:21138574

4. Andrews G, Titov N. Hit and miss: innovation and the dissemination of evidence based psychological treatments. Behav Res Ther. 2009;47(11):974–979. doi:[10.1016/j.brat.2009.07.007](https://doi.org/10.1016/j.brat.2009.07.007). PMID:19625014

5. Gega L, Marks I, Mataix-Cols D. Computer-aided CBT self-help for anxiety and depressive disorders: experience of a London clinic and future directions. J Clin Psychol. 2004;60(2):147–157. doi:[10.1002/jclp.10241](https://psycnet.apa.org/doi/10.1002/jclp.10241). PMID:14724922

6. Titov N. Status of computerized cognitive behavioural therapy for adults. Aust N Z J Psychiatry. 2007;41(2):95–114. doi:[10.1080/00048670601109873](https://doi.org/10.1080/00048670601109873). PMID:17464688

7. Waller R, Gilbody S. Barriers to the uptake of computerized cognitive behavioural therapy: a systematic review of the quantitative and qualitative evidence. Psychol Med. 2009 May;39(5):705–712. doi:10.1017/S0033291708004224. PMID:18812006
