# Supplementary material for: Views of Implementers and Nonimplementers of Internet-Administered Cognitive Behavioral Therapy for Depression and Anxiety: Survey of Primary Care Decision Makers in Sweden
Source: J Med Internet Res. 2020 Aug 12;22(8):e18033. doi: 10.2196/18033 (PMC7450364; doi:10.2196/18033)
Supplement: Multimedia Appendix 3 [file jmir_v22i8e18033_app3.docx]

## Appendix 3. Online questionnaire

Welcome to the study about introduction of internet-based cognitive behavioral therapy (ICBT) in primary care! The aim of the study is to describe the organizations that have introduced and not introduced ICBT respectively and identify possible explanations to the outcomes concerning introduction/non-introduction. By participating you can provide an important contribution to our research and development of knowledge concerning the introduction of ICBT. The questionnaire will take approximately 10-15 minutes to fill in. We are interested about your opinions and thus there are no right or wrong answers.

If you have any questions about the study or the questionnaire please do not hesitate to contact X at Y or through telephone: XX

We appreciate your effort in filling in the questionnaire.

Please click “Next” to begin the questionnaire.

# 1. Please provide the participation number you have previously received through the invitation.

___________

# Below you will find questions about you and your organization. Please choose the alternative that best describes you and your organization. You can provide multiple answers on certain questions.

# 2. What is the name of your organization and in which municipality is it located in?

# 3. What is your position in the organization?

Director of the primary care unit/Someone else, what position? – please provide details.

# 4. Is your organization a private or a public organization?

Private/Public

# 5. What is your profession? Multiple answers are allowed.

Medical doctor specialized in psychiatry/AT or ST doctor specialized in other field than psychiatry/Registered Psychologist/PTP psychologist/Nurse/Mental health nurse/Mental health worker/Social worker/Registered Psychotherapist/Something else, what? – please describe.

# 6. Does your organization offer face-to-face cognitive behavioral therapy (CBT) for adults with depression and/or anxiety?

Yes/No (In case selecting “No” you will be moved to another question)

# 7. In what way have you received information about CBT as a treatment method for adults with depression and/or anxiety? Multiple answers are allowed.

Scientific journals/Books/conferences/lectures/education/I do not have any knowledge in CBT as a treatment method for adults with depression and/or anxiety/otherwise, please describe.

# 8. How many people in your organization work with face-to-face CBT to treat adults with depression and/or anxiety?

1-2,3-4,5-10,11-20,>20

# 9. What are the professional backgrounds of the persons, in your organization, who work with face-to-face CBT to treat adults with depression and/or anxiety?

MD specialized in psychiatry/AT or ST doctor specialized in other field than psychiatry/Registered Psychologist/PTP psychologist/Nurse/Mental health nurse/Mental health worker/Social worker/Registered Psychotherapist/Something else, what? – please provide details.

# 10. Has your organization ever offered internet-based CBT (ICBT) to adults with depression and/or anxiety?

Yes/No

# 11. Does your organization refer patients to other organizations who provide ICBT to adults with depression and/or anxiety?

Yes/No

# 12. Does your organization currently offer ICBT programs to adults with depression and/or anxiety?

Yes/No (In case selecting “No” you will be moved to another question)

# 13. How has your organization received access to the ICBT programs?

We have bought the programs/We have developed the programs/Some other way, how?

# 14. If you have bought the programs, from whom have you bought the programs targeting adults with depression and/or anxiety?

Don’t know/From an organization, please provide the name of the provider_______________

# 15. Who took the initiative to introduce the ICBT programs in your organization, targeting adults with depression and/or anxiety? Multiple answers are allowed.

Director of the primary care unit/CBT therapist/Responsible for psychiatry/Head of doctors/Someone else, please indicate the position of the person

# 16. Who, at your organization, is responsible for providing ICBT programs targeting adults with depression and/or anxiety?

Director of the primary care unit/CBT therapist/Responsible for psychiatry/Head of doctors/Someone else, please indicate the position of the person

# 17. How many people, in your organization, work with the ICBT programs to treat adults with depression and/or anxiety?

1-2,3-4,5-10,11-20,>20

# 18. In your organization, what are the professional backgrounds of the persons who work with the ICBT programs to treat adults with depression and/or anxiety?

MD specialized in psychiatry/AT or ST doctor specialized in other field than psychiatry/Registered Psychologist/PTP psychologist/Nurse/Mental health nurse/Mental health worker/Social worker/Registered Psychotherapist/Something else, what? – please provide details.

# 19. Do you require that the persons in your organization, who work with the ICBT programs to treat adults with depression and/or anxiety, have been trained in CBT?

Yes/No

# 20. How many adults with depression and/or anxiety in your organization suffering from depression and/or anxiety have been treated with ICBT programs during the last 12 months?

1-10,11-30,31-50,51-100,101-200,201-400,401-1000,>1000

# 21. How do adults with depression and/or anxiety get access to the ICBT programs in your organization?

Self-referral by the patients/Referral from the general practitioner/Some other way, how? (Please describe)

# Below you will find a set of statements. Please indicate, based on your judgement, the extent to which you agree/disagree with each statement. 1 = I strongly disagree and 7 = I strongly agree. In case needed you can even choose one of the options; “Do not know” or “Do not wish to answer.” Please note that there are no right or wrong answers.

22. Adults with depression and/or anxiety have the computer skills needed to use ICBT programs (1-2-3-4-5-6-7) Do not know, Do not wish to answer.

23. Adults with depression and/or anxiety are capable of working on their own with ICBT programs (1-2-3-4-5-6-7) Do not know, Do not wish to answer.

24. Adults with depression and/or anxiety have interest in ICBT programs (1-2-3-4-5-6-7) Do not know, Do not wish to answer.

25. Adherence to treatment increases when the treatment is delivered through internet to adults with depression and/or anxiety (1-2-3-4-5-6-7) Do not know, Do not wish to answer.

26. The barrier to seek help, for adults with depression and/or anxiety, is decreased when care is provided through internet (1-2-3-4-5-6-7) Do not know, Do not wish to answer.

27. Adults with depression and/or anxiety prefer to give confidential information to a computer rather than to a person who is a CBT therapist (1-2-3-4-5-6-7) Do not know, Do not wish to answer.

28. Adults with depression and/or anxiety in rural areas can be reached with the help of ICBT programs (1-2-3-4-5-6-7) Do not know, Do not wish to answer.

# Below you will find a set of statements. Please indicate, based on your judgement, the extent to which you agree/disagree with each statement. 1 = I strongly disagree and 7 = I strongly agree. In case needed you can even choose one of the the options; “Do not know” or “Do not wish to answer.” Please notice that there are no right or wrong answers.

29. CBT therapists’ who treat adults with depression and/or anxiety are positive towards the ICBT programs (1-2-3-4-5-6-7) Do not know, Do not wish to answer.

30. CBT therapists’ who treat adults with depression and/or anxiety have knowledge of the ICBT programs (1-2-3-4-5-6-7) Do not know, Do not wish to answer.

31. CBT therapists’ who treat adults with depression and/or anxiety only need little training to be able to work with the ICBT programs (1-2-3-4-5-6-7) Do not know, Do not wish to answer.

32. CBT therapists’ who treat adults with depression and/or anxiety have the computer skills needed to work with the ICBT programs (1-2-3-4-5-6-7) Do not know, Do not wish to answer.

33. CBT therapists’ who treat adults with depression and/or anxiety have confidence in the guidelines recommending that ICBT programs are offered to adults with depression and/or anxiety (1-2-3-4-5-6-7) Do not know, Do not wish to answer.

34. CBT therapists’ who treat adults with depression and/or anxiety can motivate adults with depression and/or anxiety to participate in the ICBT programs (1-2-3-4-5-6-7) Do not know, Do not wish to answer.

35. CBT therapists who treat adults with depression and/or anxiety and support the introduction of the ICBT programs are more than those who oppose it (1-2-3-4-5-6-7) Do not know, Do not wish to answer.

# Below you will find a set of statements. Please indicate, based on your judgement, the extent to which you agree/disagree with each statement. 1 = I strongly disagree and 7 = I strongly agree. In case needed you can even choose one of the the options; “Do not know” or “Do not wish to answer.” Please notice that there are no right or wrong answers.

36. ICBT programs targeting adults with depression and/or anxiety should come with support/help desk for the CBT therapists (1-2-3-4-5-6-7) Do not know, Do not wish to answer.

37. ICBT programs are well suited for adults with depression and/or anxiety (1-2-3-4-5-6-7) Do not know, Do not wish to answer.

38. ICBT programs targeting adults with depression and/or anxiety offer alternative learning formats for those who prefer something else than pure text (1-2-3-4-5-6-7) Do not know, Do not wish to answer.

39. ICBT programs targeting adults with depression and/or anxiety are not plagued with big technical problems (1-2-3-4-5-6-7) Do not know, Do not wish to answer

40. It should be possible to trial the ICBT programs targeting adults with depression and/or anxiety before starting to use them (1-2-3-4-5-6-7) Do not know, Do not wish to answer.

41. It is possible to measure the effect on depression and/or anxiety when providing treatment through the ICBT programs for adults with depression and/or anxiety (1-2-3-4-5-6-7) Do not know, Do not wish to answer.

42. ICBT programs targeting adults with depression and/or anxiety are easy to use for the CBT therapists (1-2-3-4-5-6-7) Do not know, Do not wish to answer.

43. ICBT programs targeting adults with depression and/or anxiety can be integrated with the existing care structure that we have in place at our organization (1-2-3-4-5-6-7) Do not know, Do not wish to answer.

44. ICBT programs targeting adults with depression and/or anxiety can replace the traditional CBT treatment where the therapist meets the patient (1-2-3-4-5-6-7) Do not know, Do not wish to answer.

45. It is easy to purchase or get access to the ICBT programs targeting adults with depression and/or anxiety (1-2-3-4-5-6-7) Do not know, Do not wish to answer.

46. The existing ICBT programs targeting adults with depression and/or anxiety are well grounded on evidence from research (1-2-3-4-5-6-7) Do not know, Do not wish to answer.

47. General practitioners referring adults with depression and/or anxiety to ICBT are positive towards the ICBT programs targeting adults with depression and/or anxiety (1-2-3-4-5-6-7) Do not know, Do not wish to answer.

# Below you will find a set of statements. Please indicate, based on your judgement, the extent to which you agree/disagree with each statement. 1 = I strongly disagree and 7 = I strongly agree. In case needed you can even choose one of the the options; “Do not know” or “Do not wish to answer.” Please notice that there are no right or wrong answers.

48. Our organization has the resources required to offer the ICBT programs to adults with depression and/or anxiety (1-2-3-4-5-6-7) Do not know, Do not wish to answer.

49. Use of ICBT programs can decrease care costs regarding treatment of adults with depression and/or anxiety (1-2-3-4-5-6-7) Do not know, Do not wish to answer.

50. Our organization’s existing information management system allows administration of adult patients with depression and/or anxiety enrolled in the ICBT programs (1-2-3-4-5-6-7) Do not know, Do not wish to answer.

51. Our organization’s existing quality assurance and patient safety systems are compatible with the requirements related to offer the ICBT programs to adults with depression and/or anxiety (1-2-3-4-5-6-7) Do not know, Do not wish to answer.

52. Our organization’s existing continuing education system of the CBT therapists’ are compatible with the training related to introduce the ICBT programs to adults with depression and/or anxiety (1-2-3-4-5-6-7) Do not know, Do not wish to answer.

53. Our organization’s internal regulations/guidelines allow introduction of the ICBT programs targeting adults with depression and/or anxiety (1-2-3-4-5-6-7) Do not know, Do not wish to answer.

54. Our organization’s existing contracts with service providers allow introduction of the ICBT programs targeting adults with depression and/or anxiety (1-2-3-4-5-6-7) Do not know, Do not wish to answer.

55. The concept of providing treatment via internet to adults with depression and/or anxiety is well established at our organization (1-2-3-4-5-6-7) Do not know, Do not wish to answer.

56. Our organization’s existing patient referral process allows the introduction of the ICBT programs targeting adults with depression and/or anxiety (1-2-3-4-5-6-7) Do not know, Do not wish to answer.

# Below you will find a set of statements. Please indicate, based on your judgement, the extent to which you agree/disagree with each statement. 1 = I strongly disagree and 7 = I strongly agree. In case needed you can even choose one of the the options; “Do not know” or “Do not wish to answer.” Please notice that there are no right or wrong answers.

57. The Swedish legislation does not hinder the introduction of ICBT programs to adults with depression and/or anxiety (1-2-3-4-5-6-7) Do not know, Do not wish to answer.

58. The public opinion supports the introduction of internet-based treatments targeting adults with depression and/or anxiety (1-2-3-4-5-6-7) Do not know, Do not wish to answer.

# 59. In your mind what are the most important barriers to introduction of ICBT programs? Please indicate at maximum 5 barriers.

_____________

# 60. In your mind what are the most important factors that facilitate introduction of ICBT programs? Please indicate at maximum 5 factors.

________________

# 61. Please leave any further comments or information here:

_________________________________

**Thank you for your time and contribution to our research!**

If you have any questions about the study please do not hesitate to contact X at XX or through telephone: Y

Please click the button “Finish” to register your answers.
