# Supplementary material for: Views of Implementers and Nonimplementers of Internet-Administered Cognitive Behavioral Therapy for Depression and Anxiety: Survey of Primary Care Decision Makers in Sweden
Source: J Med Internet Res. 2020 Aug 12;22(8):e18033. doi: 10.2196/18033 (PMC7450364; doi:10.2196/18033)
Supplement: Multimedia Appendix 4 [file jmir_v22i8e18033_app4.docx]

| Appendix 4. Characteristics of the study sample and organizational characteristics (n=404). | | | | | |
| --- | --- | --- | --- | --- | --- |
| Characteristic | | | |  | Value |
| Organizational position of the respondent, n (%) | | | |  |  |
|  | Healthcare center director | | | | 363(89.9) |
|  | Head of activities | | | | 21(5) |
|  | Other^a^ | | | | 20(5) |
| Organizational form, n (%) | | | |  |  |
|  | Public | | | | 273(67.6) |
|  | Private | | | | 131(32.4) |
| Profession of the respondent (top three), n (%) | | | |  |  |
|  | Nurse, district nurse, psychiatric nurse | | | | 250(61.9) |
|  | GP specialized in general medicine | | | | 62(15) |
|  | Other^b^ | | | | 62(15) |
| Organization provides CBT, n (%) | | | |  |  |
|  | Yes | | | | 363(89.9) |
|  | No | | | | 41(10.1) |
| Sources for decision makers knowledge of CBT (top five), n (%)^c^ | | |  | | |
|  | Lectures | | | | 219(60.3) |
|  | Scientific journals | | | | 172(47.4) |
|  | Education | | | | 154(42.4) |
|  | Conferences | | | | 121(33.3) |
|  | Books | | | | 111(30.6) |
| Number of persons delivering CBT at the organization, n (%) | | | |  |  |
|  | 1-2 | | | | 236(65) |
|  | 3-4 | | | | 83(23) |
|  | 5-10 | | | | 35(10) |
|  | 11-20 | | | | 6(2) |
|  | >20 | | | | 3(1) |
| Profession of the person providing CBT (top three), n (%)^c^ | | |  | | |
|  | Psychologist (including intern psychologist) and psychotherapist | | | | 360(99.2) |
|  | Social worker | | | | 156(43) |
|  | Nurse and psychiatric nurse | | | | 77(21.2) |
| Organization has tried ICBT, n (%) | | | |  |  |
|  | Yes | | | | 122(33.6) |
|  | No | | | | 241(66.4) |
| Organization refer patients to other organizations who offer ICBT, n (%) | |  | | |  |
|  | Yes | | | | 79(21.8) |
|  | No | | | | 284(78.2) |
| Organization offers ICBT, n (%) | | | | |  |
|  | Yes | | | | 83(20) |
|  | No^d^ | | | | 321(79.5) |
| Organization provides both ICBT and CBT, n (%) | | | |  |  |
|  | Yes | | | | 83 (100) |
|  | No | | | | 0 (0) |
| Organization has access to ICBT through, n (%) | | | | | |
|  | Bought the program | | | | 32(39) |
|  | Region | | | | 22(26) |
|  | Pilot project | | | | 10(12) |
|  | Some other way^e^ | | | | 10(12) |
|  | Don’t know | | | | 5(6) |
|  | Program developed by the organization | | | | 4(5) |
| Knowledge about the provider of the program in case it was bought from an organization, n (%) | | | |  |  |
|  | Don’t know who the provider is | | | | 22(69) |
|  | Know who the provider is | | | | 10(31) |
| Initiative to provide ICBT at the organization (top three), n (%) | | | |  |  |
|  | CBT therapist | | | | 47(57) |
|  | Healthcare center director | | | | 21(25) |
|  | External party (i.e., county council or region) | | | | 15(18) |
| Person responsible for providing ICBT (top three), n (%)^c^ | | | |  |  |
|  | CBT therapist | | | | 71(85) |
|  | Healthcare center director | | | | 15(18) |
|  | General practitioner | | | | 8(10) |
| Number of persons delivering ICBT, n (%) | | | |  |  |
|  | 1-2 | | | | 61(73) |
|  | 3-4 | | | | 14(17) |
|  | 5-10 | | | | 6(7) |
|  | 11-20 | | | | 2(2) |
| Profession of persons delivering ICBT (top three), n (%)^c^ | | | |  |  |
|  | Psychologist (including intern psychologist) and psychotherapist | | | | 67(81) |
|  | Social worker | | | | 31(37) |
|  | Nurse and psychiatric nurse | | | | 16(19) |
| Organization require that persons delivering ICBT at their organization are trained in ICBT, n (%) | | | |  |  |
|  | Yes | | | | 59(71) |
|  | No | | | | 24(29) |
| Number of treated patients with ICBT during the last 12 months, n (%) | | | |  |  |
|  | 1-10 | | | | 54(65) |
|  | 11-30 | | | | 17(20) |
|  | 31-50 | | | | 7(8) |
|  | 51-100 | | | | 3(4) |
|  | 101-200 | | | | 1(1) |
|  | 201-400 | | | | 1(1) |
| Patients get access to ICBT through, n (%) | | | |  |  |
|  | Referral by the therapist | | | | 36(43) |
|  | Referral by the GP | | | | 25(30) |
|  | Self-referral by the patient | | | | 14(17) |
|  | Some other way^f^ | | | | 8(10) |
| ^a^ This category includes positions such as deputy of operations, section director, CEO, deputy CEO and unit director.  ^b^ This category includes professions such as administrator, physician secretary, economist, pharmacist, chiropractor, sociologist and family physician.  ^c^ Several alternatives possible, which mean that percentages go beyond 100.  ^d^ 41 organizations did not offer CBT and thus they were not asked whether they offered ICBT as the assumption was that they would not offer ICBT. 41 organizations equal to 10.1% of the organizations and these are included in the “No” answers.  ^e^ Several different means were suggested such as through a psychologist, through Platform for Support and Care, through a contract with a third party, and through physicians who inform the patient that programs are available online. ^f^ Several different means were suggested such as a combination of self-referral and referral by the general practitioner, telephone contact with the primary care organization, both social workers and general practitioners can refer, and through drop-in. | | | | | |
